# Klinische Umsetzbarkeit der kognitiven Screeningbatterie BICAMS bei Patienten mit Multipler Sklerose: Ergebnisse der Machbarkeitsstudie in Deutschland

**DOI:** 10.1007/s00115-021-01073-5

**Published:** 2021-02-17

**Authors:** Iris﻿-﻿Katharina Penner, Melanie Filser, Sharon Jean Bätge, Alina Renner, Sebastian Ullrich, Christoph Lassek

**Affiliations:** 1 COGITO Zentrum für Angewandte Neurokognition und Neuropsychologische Forschung, Düsseldorf, Deutschland; 2 grid.411327.2 0000 0001 2176 9917 Medizinische Fakultät, Klinik für Neurologie, Heinrich-Heine-Universität, Düsseldorf, Deutschland; 3 grid.411327.2 0000 0001 2176 9917 Institut für Experimentelle Psychologie, Heinrich-Heine-Universität, Düsseldorf, Deutschland; 4 grid.461736.1 0000 0001 2183 2023 .05 Statistikberatung, Life Science Center, Düsseldorf, Deutschland; 5 Neurologische Gemeinschaftspraxis, Kassel/Vellmar, Deutschland

**Keywords:** Kognition, Erfassen kognitiver Störungen, Chronisch entzündliche Erkrankung, Kognitive Neurologie, Neuropsychologie, Cognition, Assessment of cognitive impairment, Chronic inflammatory disease, Cognitive neurology, Neuropsychology

## Abstract

**Hintergrund:**

Patienten mit Multipler Sklerose (MS) leiden in 40–70 % der Fälle unter kognitiven Störungen. Der kognitive Status gilt erwiesenermaßen als Prädiktor für Berufsfähigkeit und frühzeitige Berentung. Eine regelmäßige Erfassung der kognitiven Leistungsfähigkeit ist somit dringend indiziert.

**Zielsetzung:**

Die deutsche Fassung der international empfohlenen BICAMS (Brief International Cognitive Assessment for Multiple Sclerosis) -Batterie wurde auf Praktikabilität in der klinischen Routine neurologischer Praxen in Deutschland multizentrisch überprüft.

**Material und Methoden:**

Medizinische Fachangestellte (MFAs) wurden hinsichtlich der Durchführung und Auswertung von BICAMS geschult. Alle ausgewerteten Testbögen wurden von unabhängigen neuropsychologischen Experten überprüft.

**Ergebnisse:**

Insgesamt 1606 BICAMS-Datensätze wurden in 65 Zentren erhoben. Von diesen konnten 1573 analysiert werden. 49,7 % der erhobenen Datensätze wurden inklusive aller Auswerteschritte korrekt durchgeführt. Bei den anderen 50,3 % fanden sich Durchführungs‑, Auswerte- oder Transformationsfehler. Nach Bereinigung der Stichprobe durch fehlerbehaftete Fälle ergaben sich Werte der Interrater-Reliabilität pro Testverfahren in Höhe von ICC $\geq$ 0,953.

**Diskussion:**

Grundsätzlich ist BICAMS für den Einsatz im klinischen Alltag sehr zu empfehlen. Es bleibt allerdings zu betonen, dass obgleich die Interrater-Reliabilität für die final bereinigte Stichprobe sehr hoch war, im Gesamtdatensatz 50,3 % Durchführungs‑, Auswerte- oder Transformationsfehler gefunden wurden. Daraus lässt sich die Notwendigkeit ableiten, nichtpsychologisches Personal noch eingehender in der Anwendung und Auswertung von BICAMS durch Experten zu schulen und zu supervidieren.

## Abstract (en)

**Background:**

Patients with multiple sclerosis (MS) suffer from cognitive impairment in 40–70% of the cases. There is evidence that the cognitive status is predictive for working ability and early retirement. Regular assessment of cognitive functionality is therefore urgently needed.

**Purpose:**

The German validation of the Brief International Cognitive Assessment for Multiple Sclerosis (BICAMS) battery was evaluated in a multicentric way with respect to its feasibility in private neurological centers across Germany.

**Methods:**

Physician assistants were trained with respect to application and scoring of BICAMS. All scored test materials were evaluated by independent neuropsychological experts.

**Results:**

A total of 1606 BICAMS datasets were collected from 65 neurological centers. Of these 1573 datasets were analyzed of which 49.7% were correctly applied and scored while mistakes in application, scoring and transformation were found in 50.3%. Interrater reliability for each subtest was found to be ICC $\geq$ 0.953 when datasets containing mistakes were excluded.

**Discussion:**

In general, BICAMS is highly recommended to be applied in standard clinical care; however, it should be emphasized that although the interrater reliability in the final sample was high, serious mistakes were found in 50.3% of cases. From these findings we conclude that nonpsychological staff have to be even more intensively trained and supervised by experts in the application and scoring of BICAMS.

## Hintergrund

Kognitive Störungen treten mit einer Prävalenz von etwa 40–70 % bei Multiple-Sklerose(MS)-Patienten auf [3], wobei die Varianz in den Angaben primär auf die verwendeten Testinstrumente und gewählten Schwellenwerte zurückzuführen ist. Die Leistungsveränderungen können bereits früh im Krankheitsgeschehen auftreten und sind auch bei einem klinisch isolierten (KIS; [15, 20]) und radiologisch isolierten Syndrom (RIS; [24]) mit sensitiven Testverfahren nachweisbar. Kognitive Störungen sind bei MS weitgehend unabhängig vom Behinderungsgrad (Expanded Disability Status Scale, EDSS; [22]), was die Bedeutsamkeit einer regelmäßigen und zuverlässigen Erfassung der kognitiven Fähigkeiten auch bei nicht oder wenig körperlich eingeschränkten Patienten nahelegt. Dies ist vor allem vor dem Hintergrund relevant, da die Arbeitsfähigkeit der Patienten eine direkte Funktion der kognitiven Leistungsfähigkeit darstellt [8, 21]. Konform dazu belegen verschiedene Studien, dass kognitiv beeinträchtigte Patienten im direkten Vergleich zu kognitiv intakten Personen nicht nur seltener berufstätig sind, sondern auch mehr Unterstützung in der Alltagsbewältigung benötigen und weniger sozial eingebunden sind [1, 14, 28].

MS-typische kognitive Veränderungen äußern sich nicht in allen Leistungsdomänen, sondern zeigen sich besonders deutlich in der kognitiven Geschwindigkeit [4, 9], dem visuell-räumlichen und sprachbezogenen Kurzzeitgedächtnis [12, 19] sowie der mentalen Flexibilität und dem Arbeitsgedächtnis [5, 11]. Die kognitive Verlangsamung gilt dabei als sog. „rote Flagge“, da sie meist die im Verlauf zuerst beeinträchtigte kognitive Domäne ist und sie – sofern sie unmittelbar nach der Diagnosestellung erhoben wird – prädiktiv für den beruflichen Status der Patienten nach mehreren Jahren sein kann [29]. Ein weiteres Argument für eine frühzeitige Erfassung des kognitiven Status bereits zum Zeitpunkt der Diagnosestellung wird durch die Ergebnisse einer longitudinalen Untersuchung gestützt [26]. Hier wurden Patienten über einen Zeitraum von acht Jahren neuropsychologisch und mittels Magnetresonanztomographie untersucht. Patienten, bei denen bereits zum Zeitpunkt der Diagnosestellung kognitive Einbußen mittels der Brief Repeatable Battery of Neuropsychological Tests (BRBN; [27]) objektiviert werden konnten, zeigten nach acht Jahren die stärkste Behinderungsprogression, konvertierten häufiger zu einer sekundär progredienten MS und wiesen eine stärkere Ausdünnung des Kortex auf. Die Ergebnisse lassen darauf schließen, dass kognitive Veränderungen, die zu Beginn der Erkrankung bereits präsent sind, zur Identifizierung von Hochrisikopatienten beitragen, bei denen eine stärkere Krankheitsprogression und damit ein aggressiverer Krankheitsverlauf zu erwarten sind. Das frühe Erfassen des kognitiven Status hat zudem nicht nur den Vorteil der klinischen Interpretation und Prädiktion, sondern ist darüber hinaus auch von entscheidender gesundheitsökonomischer Bedeutung. Neueste Zahlen zu den Kosten der MS in Europa belegen, dass kognitive Defizite unabhängig vom Behinderungsgrad auftreten und sie an den Krankheitskosten einen erheblichen Anteil haben [21]. Vergleichbare Ergebnisse wurden für Deutschland, die Schweiz und für zahlreiche andere europäische Länder vorgelegt, sodass diese Zahlen als verlässlich anzusehen sind [10, 17].

Aufgrund der besonderen Relevanz kognitiver Einschränkungen hat ein internationales Expertengremium die Verwendung einer neuropsychologischen Screeningbatterie (Brief International Cognitive Assessment for Multiple Sclerosis, BICAMS; [23]) empfohlen, um die klinische Versorgung von Patienten mit MS zu verbessern. Die Durchführung nimmt ca. 15–20 min in Anspruch und soll auch in kleineren Einrichtungen und Praxen ohne spezielle neuropsychologische Expertise anwendbar sein [23]. Die deutschsprachige Validierung der BICAMS-Testbatterie wurde bereits 2018 veröffentlicht [16].

Das Ziel der vorliegenden Studie bestand darin, den Einsatz von BICAMS in der klinischen Routine neurologischer Praxen in Deutschland, multizentrisch hinsichtlich Praktikabilität zu überprüfen und die Frage zu beantworten, ob BICAMS von nichtpsychologisch ausgebildetem Personal ordnungsgemäß durchgeführt und ausgewertet werden kann.

## Methode

### Design

Medizinische Fachangestellte (MFAs) in niedergelassenen, neurologischen Arztpraxen in ganz Deutschland wurden nach einem standardisierten Protokoll von einem neuropsychologischen Experten hinsichtlich der Durchführung und Auswertung von BICAMS „face-to-face“ geschult. Im Anschluss wurde der Schulungserfolg anhand von Rollenspielen an gesunden Personen überprüft, sodass der Experte den gesamten Ablauf von der Instruktion, über die Testdurchführung bis hin zur Auswertung beobachten und Rückmeldung geben konnte.

Die anonymisierten Untersuchungsunterlagen wurden postalisch an das COGITO-Zentrum in Düsseldorf übersendet, wo sie im Hinblick auf eine korrekte Durchführung und Auswertung von zwei unabhängigen neuropsychologischen Experten gemäß des Vier-Augen-Prinzips nach GCP („good clinical practice“) evaluiert wurden. Des Weiteren wurde die Übereinstimmung zwischen der Auswertung der MFAs und der Auswertung durch die neuropsychologischen Experten (Expertenrating) überprüft.

Ein positives Votum der Ethikkommission der Medizinischen Fakultät der Heinrich Heine Universität Düsseldorf wurde ordnungsgemäß eingeholt (Studiennummer 6035).

### Zusammensetzung der BICAMS-Screeningbatterie

Die BICAMS-Batterie beinhaltet drei Testverfahren, die jene kognitiven Domänen abbilden, die bei MS-Patienten häufig beeinträchtigt sind. Um zeitlich im Bereich einer Screeningbatterie zu bleiben (15–20 min), wurde beim VLMT und BVMT‑R ausschließlich auf die Lerntrials fokussiert. Im Folgenden werden die drei Testverfahren kurz vorgestellt:

Beim *Symbol Digit Modalities Test* (SDMT; [30]) werden den Patienten verschiedene Symbole präsentiert, die anhand einer Vorlage jeweils den Zahlen von 1 bis 9 zugeordnet werden sollen. Diese Zahl soll laut genannt werden. Die Bearbeitungszeit beträgt 90 s. Gemessen werden mit diesem Test die Informationsverarbeitungsgeschwindigkeit und das Arbeitsgedächtnis. Der SDMT hat sich als sensitiver, zuverlässiger und aufgrund der Zeitökonomie für den klinischen Alltag als sehr praktikabler Test erwiesen und gilt derzeit als der wichtigste neuropsychologische Test zur Charakterisierung der Prozessierungsgeschwindigkeit bei Patienten mit MS.

Der *California Verbal Learning Test II* (CVLT-II; [13]) und der *Verbale Lern- und Merkfähigkeitstest* (VLMT; [18]) sind Untersuchungsverfahren, mit denen das verbale Kurzzeitgedächtnis und Lernen untersucht werden. Beim CVLT-II werden 16 Wörter vorgelesen, von denen je vier zu einer übergeordneten Kategorie gehören (z. B. Kleidungsstücke, Gemüse). Die Patienten sollen so viele Wörter wie möglich sofort nach der Präsentation wieder abrufen. Es gibt insgesamt fünf Durchgänge, in denen den Patienten jeweils alle Begriffe wiederholt vorgelesen werden. Der VLMT ist ähnlich aufgebaut mit dem Unterschied, dass nur 15 Wörter präsentiert werden, die aber nicht zu einer übergeordneten Wortkategorie gehören.

Der *Brief Visual Memory Test-Revised* (BVMT‑R; [7]) erfasst das visuell-räumliche Kurzzeitgedächtnis und Lernen. Den Patienten wird für 10 s eine Vorlage mit sechs geometrischen Formen präsentiert. Danach sollen diese Formen und ihre Lokalisation aus dem Gedächtnis so genau wie möglich auf ein leeres Blatt Papier gezeichnet werden. Es gibt insgesamt drei Durchgänge, während derer wiederholt die gleichen Formen gezeigt werden und die Patienten eine neue Zeichnung anfertigen sollen.

### Statistisches Vorgehen

Die Angaben aus den erhaltenen Protokollbögen wurden in SPSS (IBM Statistical Package for the Social Sciences, Version 25.0.0) übertragen und mithilfe einer doppelten Dateneingabe und anschließendem Abgleich für potenzielle Eingabefehler korrigiert. Die statistische Auswertung fokussierte zum einen auf Fehler in der Durchführung und Auswertung von BICAMS sowie auf die Übereinstimmung zwischen der Auswertung seitens der MFAs und der neuropsychologischen Experten. Da die Übereinstimmung zwischen den beiden Experten hoch war (ICCs ≥ 0,995) wurde der Mittelwert beider Bewertungen in der Folge als „ein Expertenrating“ gewählt. Auf diese Weise wurde die in der klinischen Praxis vorkommende geringe Varianz zwischen Experten realistisch abgebildet.

### Schritte zur Überprüfung der Testdurchführung und Auswertung

Zunächst wurden die eingegangenen Untersuchungsunterlagen auf mögliche Testabbrüche seitens der Patienten geprüft.

In einem zweiten Schritt wurden die verbliebenen Fälle eingehend qualitativ auf ersichtliche Durchführungsfehler evaluiert. Als Durchführungsfehler wurden u. a. definiert: Verwendung einer falschen Stimulusvorlage, inkorrekte Verwendung der richtigen Vorlage (z. B. BVMT-R-Vorlage anders orientiert) oder die schriftliche Durchführung des SDMTs.

In einem dritten Schritt wurde die Qualität der Auswertung für die verbliebenen Fälle ohne ersichtliche Durchführungsfehler überprüft. Auswertefehler beinhalteten alle Fälle mit entweder einem fehlenden oder einem ungültigen Rohwert (d. h. Rohwerte außerhalb des möglichen Wertebereiches des jeweiligen Tests).

Zusätzlich wurde die Überführung der Testrohwerte in entsprechende alters- und bildungskorrigierte Transformationswerte des jeweiligen Tests überprüft. Konkret wurden die Transformationsfehler als eine inkorrekte Überführung der Rohwerte in z‑Werte (SDMT) bzw. Prozentränge (VLMT und BVMT-R) anhand der entsprechenden Manuale definiert.

### Übereinstimmung der Testwerte mit dem Ergebnis der Experten

Zur Überprüfung der Interrater-Reliabilität wurden die ermittelten Rohwerte der MFAs, die innerhalb des möglichen Testranges lagen, hinsichtlich der absoluten Übereinstimmung mit dem Expertenurteil überprüft.

Um der Frage nachzugehen, ob die ermittelten Differenzen zwischen Experten und MFAs klinisch relevant waren, wurden Grenzwerte für eine klinische Bedeutsamkeit pro Testverfahren angelegt. Die Angaben zu den Grenzwerten variieren in der Literatur sehr stark. Ein valides Außenkriterium zur Bestimmung der jeweiligen Grenzwerte ist die Angabe zum Arbeitsstatus der Patienten. In einer neueren Arbeit von Benedict und Kollegen wurde die Arbeitsfähigkeit als Außenkriterium zur Einstufung der kognitiven Leistung genutzt [8]. Die hieraus ableitbaren Grenzwerte der einzelnen Tests zur Unterscheidung zwischen nichtarbeitenden und arbeitenden Patienten wurden für die folgenden Analysen genutzt: SDMT ≥ 15 Punkte; VLMT ≥ 8 Punkte; BVMT ≥ 6 Punkte.

In einem letzten Schritt wurde zur Quantifizierung der Übereinstimmung zwischen den Beurteilern (Interrater-Reliabilität) der Intraklassenkoeffizient (ICC) berechnet. Dabei wurde das Expertenrating hinsichtlich der ermittelten Rohwerte mit dem der MFAs verglichen.

**Figure 1:**
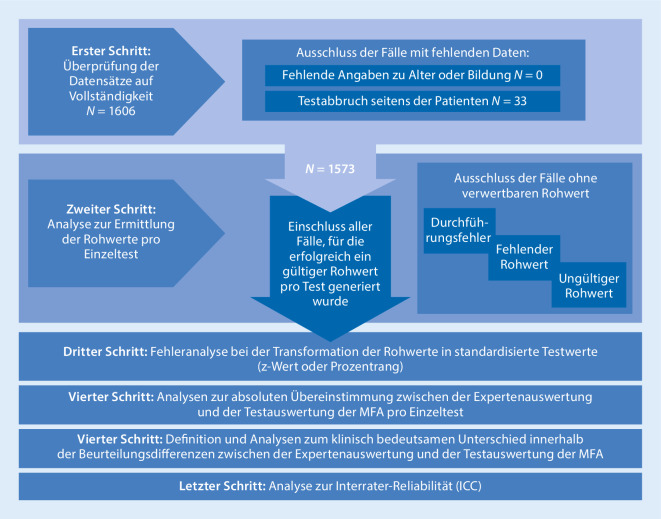

Eine Übersicht zum methodischen Vorgehen und den einzelnen Analyseschritten ist in Abb. 1 dargestellt.

## Ergebnisse

Insgesamt nahmen 65 neurologische Praxen in Deutschland an der Studie teil, von denen 1606 BICAMS-Datensätze erhoben wurden. Diese wurden zunächst auf Vollständigkeit überprüft. Durch Testabbrüche seitens der Patienten mussten 33 (2,1 %) Datensätze von der weiteren Analyse ausgeschlossen werden, sodass für alle weiteren Analysen 1573 Datensätze berücksichtigt werden konnten.

Die demografischen Angaben der verbleibenden Stichprobe (*n* = 1573) sind Tab. 1 zu entnehmen.

**Table 1:** 

	MS-Patienten *n* = 1573
*Geschlecht: weiblich (%)*	*73,1*
*Alter in Jahren (M [SD])*	*43 (11,29)*
*Krankheitsverlauf (n [%])*	
RRMS	1350 (89,94)
SPMS	114 (7,60)
PPMS	37 (2,46)
*Erkrankungsdauer in Jahren (M [SD])*	9,66 (7,81)
*Schulbildung (%)*	
Hauptschulabschluss	20,9
Mittlere Reife	46,4
Abitur	32,7
*Berufstätigkeit (%)*	
Nicht berufstätig	32,5
In Teilzeit berufstätig	24,0
In Vollzeit berufstätig	40,2
*Motorik (%)*	
Keine Einschränkungen	55,6
Funktionelle Einschränkungen	10,8
Gehfähigkeit vermindert	30,8

Hinweis: nicht vorliegende Informationen: Geschlecht *n* = 6, Erkrankungsdauer* n* = 79, Krankheitsverlauf *n* = 72, Berufstätigkeit *n* = 51, Motorik *n* = 45

### Durchführungs- und Auswertefehler (Ermitteln der Testrohwerte) pro Einzeltest

Beim SDMT wurden bei 179 Fällen (11,4 %) Fehler beim Bestimmen des Rohwertes (d. h. durch eine falsche Durchführung, durch eine nicht stattgefundene Auswertung oder durch das Bestimmen eines Rohwertes, der nicht dem gültigen Wertebereich des jeweiligen Testmanuals entspricht) gefunden, beim VLMT bei 130 Fällen (8,3 %) und beim BVMT‑R bei 219 Fällen (13,9 %).

Bezogen auf die Gesamtbatterie BICAMS wurden 1162 (73,49 %) Datensätze von den Experten als korrekt durchgeführt beurteilt. Bei 411 (26,1 %) Datensätzen kam es zu diversen Fehlern in mindestens einem der drei Subtests.

### Transformationsfehler (Ermitteln von z-Werten und Prozenträngen)

Bei der Transformation der ermittelten Rohwerte in z‑Werte oder Prozentränge anhand der vorliegenden Handbücher zeigte sich in Abhängigkeit vom jeweiligen Testverfahren eine unterschiedlich stark ausgeprägte Anfälligkeit für Fehler.

Beim SDMT zeigten sich in den nach Abzug der 179 fehlerbehafteten Rohwertfälle (*n* = 1394) bei 128 Fällen (9,2 %) Fehler bei der Transformation in einen z‑Wert. Beim VLMT wurden bezogen auf die verwertbaren Fälle (*n* = 1443) bei 442 (30,6 %) Datensätzen Fehler bei der Transformation in einen Prozentrang gefunden. Beim BVMT‑R lag die Rate der falsch transformierten Werte bei 41 Fällen (3 %).

**Figure 2:**
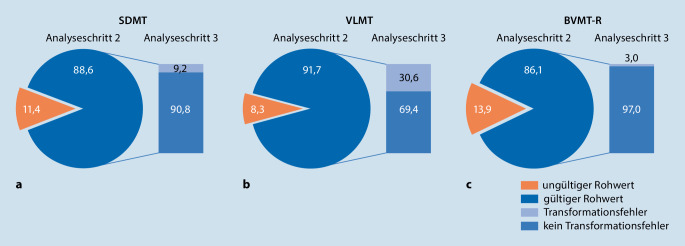

Die Abb. 2 verdeutlicht graphisch die Fehlerarten und Fehlerhäufigkeiten pro Einzeltest.

### Gesamtanteil der Fehler in Auswertung, Durchführung und Transformation der einzelnen Subtests und der Gesamtbatterie BICAMS

Da Fehler in der Durchführung, der Auswertung und/oder der Transformation der Rohwerte allesamt zu einer falschen Einschätzung der Leistungsfähigkeit der Patienten beitragen, wurde die Gesamtfehlerquote pro Einzeltest anhand der auswertbaren Gesamtstichprobe (*n* = 1573) bestimmt. Hier ergab sich für den SDMT eine Fehlerquote von 19,52 %, beim VLMT von 36,6 % und beim BVMT‑R von 16,5 %. Bezogen auf die Gesamtbatterie wurden in 781 Fällen (49,7 %) alle drei Tests korrekt durchgeführt, ausgewertet und transformiert.

### Absolute Ergebnisdifferenzen zwischen Experten und den MFAs

**Figure 3:**
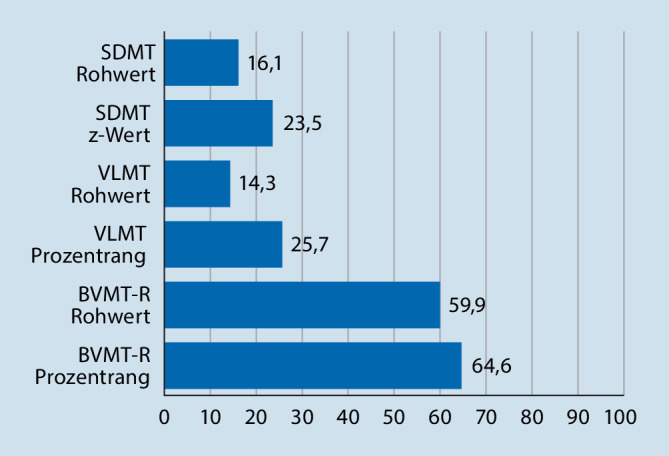

In einem weiterführenden Analyseschritt wurde analysiert, inwieweit die ermittelten Ergebnisse der MFAs mit denjenigen der Experten übereinstimmen. Für den SDMT-Rohwert zeigte sich in 16,1 % der Fälle eine Abweichung zwischen Expertenresultat und dem der MFAs. Beim Transformieren des Rohwertes in einen z‑Wert zeigte sich eine Abweichung in 23,5 % der Fälle. Für das Bestimmen des VLMT-Rohwertes gab es bei 14,3 % der Fälle Abweichungen. Beim Bestimmen des VLMT-Prozentranges gab es in 25,7 % Abweichungen zum Expertenrating. Für den BVMT‑R zeigte sich in 59,9 % der Fälle eine Abweichung beim Bestimmen des Rohwertes. Für die Transformation des BVMT-R-Rohwertes in einen Prozentrang gab es in 64,6 % der Fälle Abweichungen zum Expertenurteil. Abb. 3 verdeutlicht graphisch die Abweichungen zwischen Experten und MFAs.

### Untersuchung der klinischen Bedeutsamkeit der Differenzen in den Ergebnissen zwischen Experten und MFAs

Bemessen an den im Methodenteil beschriebenen Grenzwerten für die einzelnen Tests unterschieden sich MFAs und Experten in ihrer Beurteilung der kognitiven Leistungsfähigkeit der Patienten bezogen auf die verwertbaren Fälle nach Bereinigung aller Durchführungs- und Auswertefehler im SDMT in 2,8 %, beim VLMT in 3,9 % und im BVMT‑R in 2,5 % der Fälle.

Da der SDMT derzeit der in der klinischen Praxis am häufigsten durchgeführte Test ist und sich hierfür ein Veränderungsgrenzwert von $\geq$3 in Anlehnung an die Arbeit von Morrow und Kollegen [25] etabliert hat, wurde für diesen Test die Differenz zwischen MFAs und Experten nochmals separat mit diesem wesentlich geringeren Grenzwert gerechnet. Hierbei ergab sich in 5,8 % der Fälle ein klinisch relevanter Unterschied.

## Diskussion

Da die Erstdiagnose der MS oftmals in einer Lebensphase gestellt wird, in der viele wichtige und prägende Entscheidungen getroffen werden, haben kognitive Veränderungen auf das soziale und berufliche Leben gravierenden Einfluss [2, 28]. Hinzu kommt eine nicht unerhebliche ökonomische Belastung für das jeweilige Gesundheitssystem [21]. Um betroffene Patienten so früh wie möglich zu detektieren, sie symptomatisch zu behandeln und gegebenenfalls die bestehende Immuntherapie zu überdenken, ist es notwendig, bereits zur Diagnosestellung den kognitiven Status zu erheben und ihn in der Folge in jährlichen Abständen zu überprüfen. Damit diese Überprüfung in die klinische Routine aufgenommen werden kann, bedarf es einer Screeningbatterie, die auch von nichtpsychologischem Personal zeiteffizient und korrekt durchgeführt werden kann. In einer kürzlich publizierten Studie konnte gezeigt werden, dass das Anwenden eines Einzeltests wie dem SDMT nicht ausreichend ist, um eine zuverlässige Aussage über den kognitiven Status von MS-Patienten zu machen, sondern die Minimalanforderung an ein Screening eine Kombination aus SDMT und BVMT‑R beinhalten sollte [6]. Auch wenn außer Frage steht, dass ein erfahrener Neurologe im Bereich der MS kognitive und emotionale Veränderungen, gerade wenn er seine Patienten gut kennt, im Gespräch erkennen kann, ist die objektive und vergleichbare Quantifizierung und die Verlaufsdokumentation, nicht nur im Sinne einer Erleichterung des kollegialen Austausches (z. B. bei Behandlerwechsel, Behandlung in zwischen ambulant und stationär wechselnden Settings), sondern auch zur argumentativen Unterstützung in berufsrechtlichen Fragen (z. B. der Berufs[un]fähigkeit) unabdingbar. Hierzu reicht der rein „klinische Blick“ nicht aus.

Da in Deutschland eine große Anzahl MS-Patienten in nichtuniversitären Einrichtungen behandelt und betreut wird, war es das Ziel der Studie, die derzeit international genutzte Screeningbatterie BICAMS auf Machbarkeit in niedergelassenen neurologischen Praxen in Deutschland zu überprüfen.

Grundsätzlich hat die umfangreiche Studie gezeigt, dass die Anwendung von BICAMS in der klinischen Routine zeitlich möglich und praktikabel ist und von den Patienten sehr gut akzeptiert wurde (nur 33 Patienten haben ihre Teilnahme abgebrochen). Die Bereitschaft seitens der Patienten an einer kognitiven Untersuchung teilzunehmen ist ein nicht zu unterschätzender Bestandteil jeder neuropsychologischen Untersuchung, der in der Vergangenheit z. B. durch Einsatz des PASAT‑3 oder gar PASAT‑2 missachtet wurde. Hier kam es v. a. bei kognitiv eingeschränkteren Patienten immer wieder zu emotionalen Einbrüchen, weswegen dieser Test nur noch in Ausnahmefällen (z. B. dringend notwendige Verlaufsdokumentation im Rahmen des Multiple Sclerosis Functional Composite [MSFC]) zum Einsatz kommen sollte.

Die MFAs zeigten ebenso von Beginn an eine große Bereitschaft und Motivation, die Untersuchungen mit den Patienten durchzuführen und waren hinsichtlich Rekrutierung und Zusendung der Unterlagen äußerst engagiert und zuverlässig. Somit ist die Bereitschaft in den Praxen gegeben, derartige Untersuchungen auch zukünftig mit den Patienten durchzuführen.

Die genaue Überprüfung der zugesandten Unterlagen ergab allerdings, dass nur knapp die Hälfte (49,7 %) fehlerfrei durchgeführt, ausgewertet und/oder in standardisierte Leistungswerte transformiert wurde. Hierbei gilt es zu beachten, dass wenn schon in der Durchführung ein Fehler passierte, dieser fehlerbehaftete Rohwert zwar im weiteren Verlauf, entsprechend des Manuals, richtig ausgewertet und transformiert werden konnte, es sich aber am Ende dennoch nicht um die korrekte Darstellung der kognitiven Leistung handelte und die Patienten folglich fälschlicherweise einen inkorrekten kognitiven Status attestiert bekamen. Somit sind Fehler an jeder Stelle des Prozesses von der Durchführung bis hin zur Transformation in ihrer Bedeutung gleichwertig und ausdrücklich zu vermeiden. Eine genaue Durchsicht der zugesandten Daten pro Zentrum ergab zudem, dass kein Zentrumseffekt vorlag, auf den die berichteten Durchführungs‑, Auswerte- oder Transformationsfehler zurückführbar wären. Die Fehler pro Test traten zwischen den Zentren unsystematisch und unabhängig von der Qualität der Durchführung und Auswertung der jeweils anderen beiden Tests auf.

Bei den absoluten Beurteilungsdifferenzen zwischen MFAs und Experten lagen die Abweichungen für SDMT und VLMT zwischen 16,1 und 25,7 %. Noch gravierender zeigte sich die Abweichung beim BVMT‑R, die für das Ermitteln des Rohwertes bei 59,9 % und für die Transformation in einen Prozentrang bei 64,6 % lag. Beim BVMT‑R kommt zum Tragen, dass die Bewertung der Leistung einen großen Ermessensspielraum seitens des Untersuchers lässt und dieser daher ausreichend Erfahrung und Routine in der Anwendung mitbringen sollte.

Als Fazit dieser Studie lässt sich festhalten, dass in deutschen neurologischen Praxen MS-Patienten mittels BICAMS von MFAs durchaus untersucht werden können und sollten. Allerdings bedarf es einer eingehenderen sowie wiederholten Schulung/Supervision, um die in dieser Studie berichteten Fehler zukünftig vermeiden zu können. In Anbetracht der knappen zeitlichen Ressourcen in der Patientenversorgung wäre es auch denkbar die Auswertung an spezialisierte Neuropsychologen oder ein Spezialzentrum zu delegieren, sodass für die Praxen nur der Aufwand von Schulung und Durchführung verbleiben würden. Im Hinblick auf die Abrechnungsmöglichkeiten, die derzeit mitnichten kostendeckend sind, wäre letzteres Szenario auch vor diesem Hintergrund ein denkbarer Weg.

Sollten MFAs im Rahmen zukünftiger Studien die BICAMS-Batterie anwenden und auswerten müssen, so wäre anzuraten, das nichtpsychologische Personal in Form eines Workshops von Experten eingehend schulen zu lassen und im Verlauf der Studie einen Supervisor einzubinden, der das Durchführungsprozedere regelmäßig auffrischt, für auftretende Fragen und Unsicherheiten seitens der MFAs zur Verfügung steht und die Auswertungen kontrolliert. In unserer Studie hat sich gezeigt, dass das einmalige Schulen offensichtlich nicht dazu ausreicht, dass sich die neuen zum Teil auch komplexen Inhalte bei den MFAs festigen. Die Analyse der Fehler pro Zentrum hat zudem sehr deutlich gezeigt, dass wenn ein Fehler begangen wurde, dieser konstant bei einer Mehrheit der von diesem Zentrum eingereichten Datensätzen zu finden war. Dies spricht gegen „Flüchtigkeitsfehler“ und für Fehler, die auftreten durch ein Unverständnis/Missverständnis in der Applikation und Auswertung. Somit erscheint es augenscheinlich, dass durch eine engere Schulung und Supervision durch einen Experten per Telefon oder Video-Chat solche Zentren stark profitieren würden und man die Korrektheit der Durchführung und Auswertung dadurch deutlich verbessern könnte.

## Fazit für die Praxis

- Kognitive Störungen bei Multipler Sklerose treten mit einer Prävalenz von 40–70 % auf und sollten daher ernst genommen werden.
- Das Erfassen kognitiver Störungen ist relevant für die Beurteilung der Arbeitsfähigkeit der Patienten und prädiktiv für die Evolution der Krankheit über die Zeit.
- Der kognitive Status sollte bei MS-Patienten zur Diagnosestellung erfolgen und danach einmal jährlich überprüft werden.
- Der rein klinische Blick reicht für die Quantifizierung sowie die Verlaufsdokumentation des kognitiven Status im Sinne des kollegialen Austausches und zur argumentativen Unterstützung in berufsrechtlichen Fragen nicht aus.
- BICAMS ist ein international anerkanntes und empfohlenes kognitives Screeninginstrument, das sich für den Einsatz in der klinischen Routine eignet, auch von nichtpsychologisch ausgebildeten Personen nach eingehender Schulung angewendet werden kann und von den Patienten sehr gut toleriert wird.
